# Junctional Adhesion Molecule-Like Protein Promotes Tumor Progression and Metastasis *via* p38 Signaling Pathway in Gastric Cancer

**DOI:** 10.3389/fonc.2021.565676

**Published:** 2021-03-11

**Authors:** Yuying Fang, Jianmin Yang, Guohong Zu, Changsheng Cong, Shuai Liu, Fei Xue, Shuzhen Ma, Jie Liu, Yuping Sun, Meili Sun

**Affiliations:** ^1^ Department of Oncology, Jinan Central Hospital, Cheeloo College of Medicine, Shandong University, Jinan, China; ^2^ Department of Oncology, Central Hospital Affiliated to Shandong First Medical University, Jinan, China; ^3^ The Key Laboratory of Cardiovascular Remodeling and Function Research, Chinese Ministry of Education, Chinese Ministry of Health and Chinese Academy of Medical Sciences, The State and Shandong Province Joint Key Laboratory of Translational Cardiovascular Medicine, Department of Cardiology, Qilu Hospital, Cheeloo College of Medicine, Shandong University, Jinan, China; ^4^ Cardiovascular Disease Research Center of Shandong First Medical University, Central Hospital Affiliated to Shandong First Medical University, Jinan, China

**Keywords:** junctional adhesion molecule-like protein (JAML), gastric cancer, p38, tumor progression, migration

## Abstract

Junctional adhesion molecule-like protein (JAML), a newly discovered junctional adhesion molecule (JAM), mediates the adhesion and migration processes of various immune cells and endothelial/epithelial cells, ultimately regulating inflammation reaction. However, its role in tumors remains to be determined. The expression of JAML was examined in gastric cancer (GC) and peritumoral tissues from 63 patients. The relationship between JAML expression and clinical characteristics was also observed. *In vitro*, GC cell migration and proliferation were assessed by wound healing assay, transwell migration assay and EdU incorporation assay. Immunohistochemical staining results showed that JAML expression level was higher in GC tissues than in peritumoral tissues. High expression of JAML in cancer tissues was associated with worse cell differentiation, local lymph node involvement, deep infiltration, and advanced stage. *In vitro*, we found that JAML silencing inhibited GC cell migration and proliferation, while JAML overexpression promoted GC cell migration and proliferation, partially *via* p38 signaling. Taken together, our study revealed a critical role for JAML to promote GC cell migration and proliferation. JAML might be a novel diagnostic biomarker and therapeutic target for GC.

## Introduction

Gastric cancer (GC) is a malignant tumor originating from the gastric mucosa epithelium, which has high morbidity and mortality in worldwide. In 2018, there were an estimated 1,000,000 new GC cases and 783,000 deaths (1, 2). The main causes of GC death are rapid proliferation, invasion, metastasis, and anti-cancer drug resistance. However, because the symptoms of early GC are inconspicuous, the advanced stage at the diagnosis is an important factor in the gastric-cancer-related mortality (3). Therefore, it is necessary to search for effective targets for screening and diagnosing GC as early as possible, thus improving prognosis.

More and more studies have shown that complex steps such as adhesion, degradation, movement and blood vessel formation promote tumor cell infiltration and metastasis. Adhesion molecules are involved in the process of tumor metastasis (4). In recent years, the role of junctional adhesion molecules (JAMs) of immunoglobulin superfamily in cancer occurrence and progression has attracted extensive attentions (5, 6). Current research has found that tumorigenesis is associated with increased levels of JAM protein expression, and increased expression of JAM is associated with poor prognosis. The mechanism may involve the enhanced ability of tumor cells to migrate to the stroma and move across the vessel wall during local infiltration and metastatic spread (5–8).

Junctional adhesion molecule-like protein (JAML) is a new member of JAMs, which includes two extracellular immunoglobulin-like domains, a transmembrane fragment and a cytoplasmic tail. JAML has been found to be expressed in cells such as neutrophils, monocytes, some T cells, and acute promyelocytic leukemia cells. JAML mediates the adhesion and migration processes of various immune cells and endothelial/epithelial cells, ultimately regulating inflammation reaction (9–12). Although it has been found that JAML plays exact roles in the process of wound healing and atherosclerosis in recent years, its role in the tumor has been poorly investigated (13, 14). For this reason, in this study, we attempted to investigate the function of JAML in GC through *in vitro* and *in vivo* experiments.

## Materials and Methods

### Human Samples

A total of 63 tissue specimens of GC from Jinan Central Hospital between 2014 and 2018 were collected, with a median age of 64 years (range: 36–88 years). There were 49 men (77.78%) and 14 women (22.22%). We analyzed the histopathological results of GC specimens using the eighth edition of AJCC/UICC (15). Each patient provided written informed consent. This study was approved by the evaluation committee of Jinan Central Hospital of Shandong University.

### Immunohistochemical Staining

We cut the paraffin sections into 4 μM slices. The antigen was repaired with sodium citrate under high temperature and pressure. The sample was incubated with 3% H_2_O_2_ solution for 10 min to reduce endogenous peroxidase activity. It was sealed with 5% goat serum and 0.2% bovine serum albumin for 30 min. Rabbit anti-JAML polyclonal antibodies (Novus Biologicals, USA, NBP2-14286) were incubated overnight. After rewarming, the second antibody was incubated for 1 h. We then performed DAB staining and then hematoxylin staining. Two independent pathologists evaluated the results of immunohistochemistry at the same time. Scores were determined according to the degree of staining and the proportion of positive cells. The intensity score represents the average staining intensity of positive cells (0 = no staining; 1 = light yellow; 2 = buffy; 3 = brown). The proportion score represents the proportion of positively stained cells (0 = 0; 1 = less than 25%; 2 = 25–50%; 3 = 50–75%; 4 = more than 75%). The final score is the product of intensity score and proportion score: high expression ≥ 4 points; low expression < 4 points.

### Cell Culture

The human GC cell lines (AGS, HGC-27 and MKN-28) were purchased from the cell resource center of Chinese Academy of Sciences (Beijing, China). HGC-27 and MKN-28 were cultured in RPMI-1640 medium (Gibco, USA) containing 10% fetal bovine serum (FBS; Gibco). AGS was cultured in F12k medium (Macgene, China) containing 10% fetal bovine serum (FBS; Gibco). The p38 inhibitor SB-203580 was purchased commercially (Selleck, Houston, TX, USA).

### Cell Transfection

JAML plasmid (GenePharma, Shanghai, China) was formed using full length human JAML cDNA linked with the pcDNA3.1(+) vector to induce JAML over-expression in cultured GC cells. According to the manufacturer’s product instructions, JAML plasmid was transfected into the cells using X-treme GENE HP Reagents (Roche, Basel, Switzerland). Cells transfected with pcDNA3.1(+) (NC) vector was used as a negative control group. Small interfering RNA against human JAML (siJAML) (GenePharma, Shanghai, China) was transfected within gastric cells to reduce JAML expression. siRNA sequences are: siJAML1, 5’-GGAAUUGUCUGUGCCACAATT-3’, 5’-UUGUGGCACAGACAAUUCCTT-3’; siJAML2, 5’-CCAGAGCACAGAAGUGAAATT-3’, 5’-UUUCACUUCUGUGCUCUGGTT-3’; siJAML3, 5’-CCAGAGCACAGAAGACAAATT-3’, 5’-UUUGUCUUCUGUGCUCUGGTT-3’; negative control (siNC), 5’-UUCUCCGAACGUGUCACGUTT-3’, 5’-ACGUGACACGUUCGGAGAATT-3’. Cell function experiments were performed after 72 h of treatment of cells with JAML plasmids or small interfering RNA. In order to ensure the continuous and effective transient transfection during the cell function test, western blot analysis was used to test the transfection efficiency at 72 h and 120 h after transient transfection.

### Western Blot Analysis

Cells were acquired and prepared in RIPA buffer (Beyotime, China), 1% protease inhibitor cocktail 1, 1% phosphate inhibitor cocktail 2, and 1% phosphate inhibitor cocktail 3 (Sigma, USA). BCA protein assay kit (Beyotime, shanghai, China) was used to determine the protein concentration. The loading volume based on the cell concentration is calculated to ensure that the total number of loaded cells in each group is consistent. The protein extract was separated by 10% SDS-PAGE and added to the polyvinylidene difluoride membrane (Millipore, Boston, MA, USA). After electrophoresis and membrane transfer, the antibody was incubated overnight. The protein was visualized using chemiluminescence (ECL Plus Western Blot Detection System; Bio-Rad, USA). ImageJ was used to measure the gray value of bands to calibrate the expression of housekeeping gene (tubulin).The antibodies used include: rabbit anti-JAML monoclonal antibody (Abcam, USA, ab183714), rabbit anti-p-ERK1/2 monoclonal antibody (Cell signaling Technology, USA, 4370), rabbit anti-ERK1/2 monoclonal antibody (Cell signaling Technology, USA, 4695), rabbit anti-p-JNK monoclonal antibody (Cell signaling Technology, USA, 4668), rabbit anti-JNK polyclonal antibody (Cell signaling Technology, USA, 9252), rabbit anti-p-p38 monoclonal antibody (Cell signaling Technology, USA, 4511), rabbit anti-p38 monoclonal antibody (Cell signaling Technology, USA, 8690), mouse anti-tubulin monoclonal antibody (Abcam, USA, ab210797). Tubulin was used as the loading control.

### Wound Healing Assay

GC cells were covered in six-well plates (Corning Incorporated, Corning, NY, USA) and were scratched after sticking to the wall. RPMI-1640 medium (Gibco, USA) was used to culture cells, and the same field of vision was taken at 0 and 48 h respectively. Each experiment was performed in triplicate.

### Transwell Migration Assay

Cell migration was measured in 24-well plates (Corning Incorporated, Corning, NY, USA) with 8μm-pore polycarbonate membranes. Cells were seeded at a density of 4 × 10^4^ cells/well in the upper chamber with serum-free RPMI-1640 medium and incubated at 37°C for migration assay. After 48 h of culture, cells were fixed and stained with crystal violet, then observed under optical microscope (Nikon). Three fields were randomly selected for cell count. Each experiment was performed in triplicate.

### Cell Proliferation Experiment

EdU (5-Ethynyl-2´-deoxyuridine) DNA cell proliferation Kit (Beyotime, Shanghai, China and RiboBio, Guangzhou, China) was chosen to determine cell proliferation. The cells after the required treatment are counted, resuspended in culture medium, and re-seeded on a 96-well plate with 4 × 10^4^ cells per well. After incubation for 12 h, 10 μM EdU was added to the cultures and 2 h later cells were collected. According to the operation requirements of the kit, after fixation, washing, penetration and dye marking, observe and take photos with fluorescence microscope (Nikon). Each experiment was performed in triplicate.

### Statistical Analysis

Statistical analysis was performed using SPSS version 20.0 (SPSS, Chicago, Illinois, USA) and GraphPad Prism 8.0 software (San Diego, CA, USA). Statistical significance was assessed by Student’s t-test between two groups or by one-way ANOVA between three or more groups for continuous data. Chi-square test was used to analyze the association between JAML expression and clinicopathological variables. Experimental data were presented as mean ± standard deviation (SD). *P* < 0.05 was considered statistically significant.

## Results

### Junctional Adhesion Molecule-Like Protein Was Highly Expressed in Human Gastric Cancer Tissues and High Junctional Adhesion Molecule-Like Protein Expression in Gastric Cancer Correlated With Advanced Clinicopathological Features

**Figure 1:**
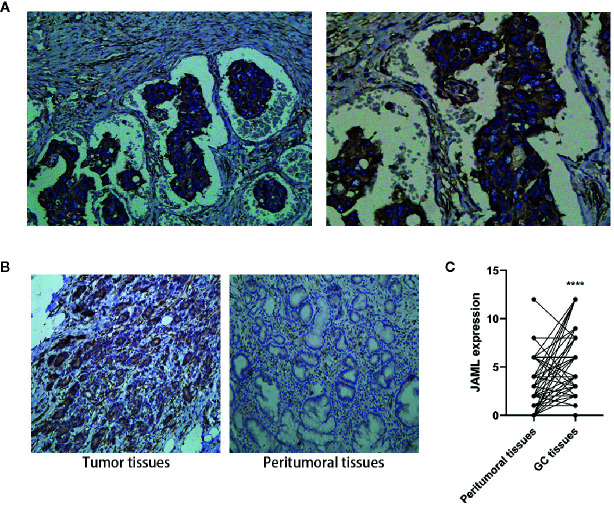
Expression of JAML in human gastric cancer (GC) and peritumoral tissues. **(A)** JAML expression on cytoplasm and membrane of GC cells. **(B)** The JAML expression in GC and peritumoral tissues. **(C)** Quantitative analysis of JAML expression in GC and peritumoral tissues. n=63, paired t test, ****P < 0.0001, compared with peritumoral tissues.

The detailed clinicopathological parameters and JAML expression of patients with gastric cancer were presented in the **Supplemental Data Sheet**. The expression of JAML in 63 cases of GC was detected by IHC, and the relationship between JAML and clinicopathological parameters was also analyzed. JAML was expressed in the cytoplasm and membrane of cancer cells (**Figure 1A**). IHC analysis showed that JAML in GC tissue was significantly up-regulated compared with peritumoral tissues (**Figures 1B, C**). Thereafter, we investigated the relationship between JAML expression and various pathological parameters in GC tissues. We found that high expression of JAML in GC cells was associated with poor cell differentiation (*P* = 0.001), local lymph node involvement (*P* = 0.012), deeper infiltration (*P* = 0.026), and advanced stages (*P* = 0.021) (**Table 1**).

**Table 1:** Correlation between JAML expression and clinicopathological parameters in human GC tissues.

Variables	JAML expression		
	high	low	p
Age (year)			
<60	12	4	0.609
≥60	30	17	
Gender			
Male	31	18	0.453
Female	11	3	
Primary tumor			
T0-T2	31	21	0.026
T3-T4	11	0	
Regional lymph node involvement			
N0-N1	18	16	0.012
N2-N3	24	5	
Histological grade			
G1-G2	11	15	0.001
G3	31	6	
TNM stage groupings			
I–II	22	18	0.021
III	20	3	

GC, gastric cancer; TNM, tumor, node, metastasis.

### Junctional Adhesion Molecule-Like Protein Promoted Gastric Cancer Cell Proliferation and Migration

**Figure 2:**
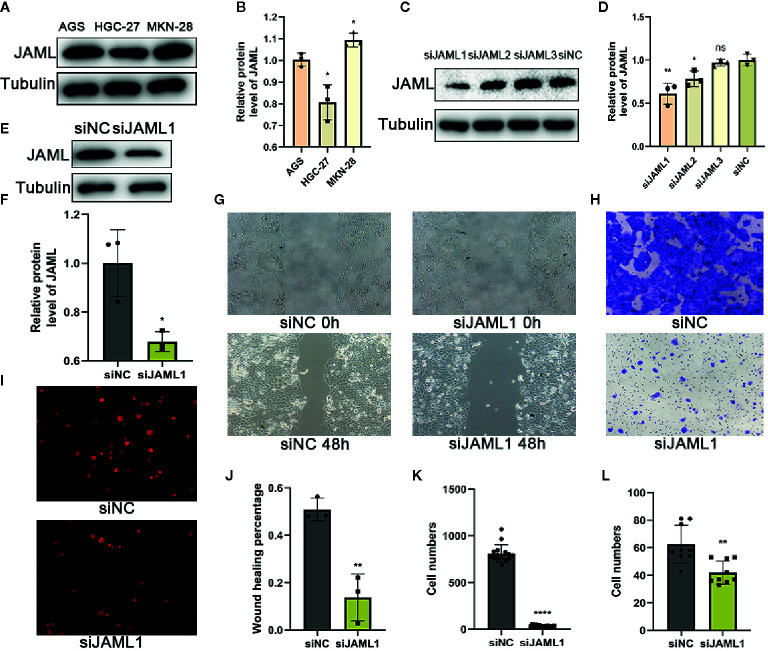
JAML promoted GC cell proliferation and migration. **(A)** The expression of JAML in GC cell lines (AGS, HGC-27, MKN-28). **(B)** Quantitative analysis of **(A)** n=3, unpaired t test, *P < 0.05, compared with AGS group. **(C)** Knockdown efficiency of JAML was confirmed in MKN-28 cells after transient transfection of SiRNA for 72h by western blot. **(D)** Quantitative analysis of **(C)** n=3, unpaired t test, **P < 0.01, compared with siNC group; n=3, unpaired t test, *P < 0.05, compared with siNC group; n=3, unpaired t test, ns, *P*>0.05, compared with siNC group. **(E)** Knockdown efficiency of JAML in MKN-28 cells after transient transfection of siRNA for 120h by western blot. **(F)** Quantitative analysis of **(E)** n=3, unpaired t test, *P < 0.05, compared with siNC group. **(G)** Wound healing assay was performed in transfected MKN-28 cells treated with or without siRNA to evaluate cell migration. **(H)** Transwell migration assay to assess cell migration. **(I)** EdU incorporation assay to observe cell proliferation. **(J)** Quantitative analysis of **(G)** n=3, unpaired t test, **P < 0.01, compared with siNC group. **(K)** Quantitative analysis of **(H)** n=15, unpaired t test, ****P < 0.0001, compared with siNC group. **(L)** Quantitative analysis of **(I)** n=9, unpaired t test, **P < 0.01, compared with siNC group. siNC, negative control.

**Figure 3:**
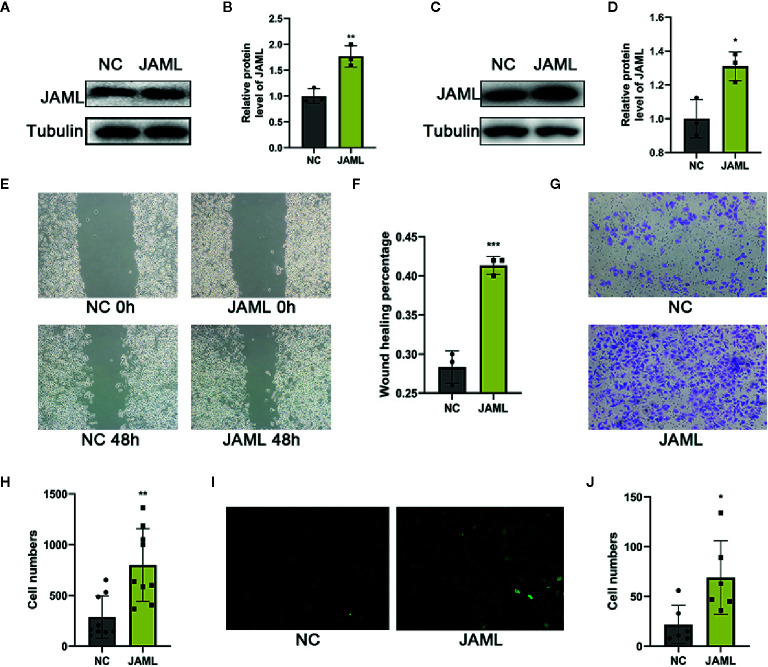
**(A)** JAML expression in HGC-27 cells after transfection with JAML plasmid for 72h. **(B)** Quantitative analysis of **(A)** n=3, unpaired t test, **P < 0.01, compared with NC group. **(C)** JAML expression in HGC-27 cells after transfection with JAML plasmid for 120h. **(D)** Quantitative analysis of **(C)** n=3, unpaired t test, *P < 0.05, compared with NC group. **(E)** Wound healing assay to assess cell migration in HGC-27 cells. **(F)** Quantitative analysis of **(E)** n=3, unpaired t test, ***P < 0.001, compared with NC group. **(G)** Transwell migration assay to evaluated cell migration in HGC-27 cells. **(H)** Quantitative analysis of **(G)** n=9, unpaired t test, **P < 0.01, compared with NC group. **(I)** EdU incorporation assay to observe cell proliferation in HGC-27 cells. **(J)** Quantitative analysis of **(I)** n=6, unpaired t test, *P < 0.05, compared with NC group. NC, negative control.

The result that high JAML levels were associated with higher tumor malignancy in GC patients encouraged us to assess whether JAML was related to oncogenic function. First, we examined JAML expression in GC cell lines (AGS, HGC-27, and MKN-28) (**Figures 2A, B**). The expression of JAML was relatively higher in MKN-28 cells, while was lower in HGC-27 cells. Thus, small interfering RNA against human JAML (siJAML) was transfected to MKN-28 cells to reduce JAML expression. The results showed that the knockdown effect of siJAML1 was the most effective (**Figures 2C, D**) and was stable for 5 days (**Figures 2E, F**), so siJAML1 was used for the subsequent experiments. The wound healing and transwell migration assays showed that JAML deficiency significantly decreased migration in MKN-28 cells (**Figures 2G, H, J, K**). In addition, the EdU incorporation assay demonstrated the proliferation of MKN-28 cells was significantly inhibited after silencing of JAML (**Figures 2I, L**). Next, we transfected JAML plasmid to HGC-27 cells to increase the expression of JAML. Western blot analysis showed that the JAML plasmid transfection up-regulated the expression of JAML in HGC-27 cells (**Figures 3A, B**), and the effect was stable until the 5th day after transfection (**Figures 3C, D**). The wound healing and transwell migration assays showed that JAML overexpression significantly increased migration in HGC-27 cells (**Figures 3E–H**). In addition, the EdU incorporation assay showed that JAML overexpression enhanced HGC-27 cells proliferation (**Figures 3I, J**). These results suggested that JAML might facilitated GC migration and proliferation.

### Junctional Adhesion Molecule-Like Protein Promoted Gastric Cancer Cell Migration and Proliferation by Activating p38 Signaling Pathway

**Figure 4:**
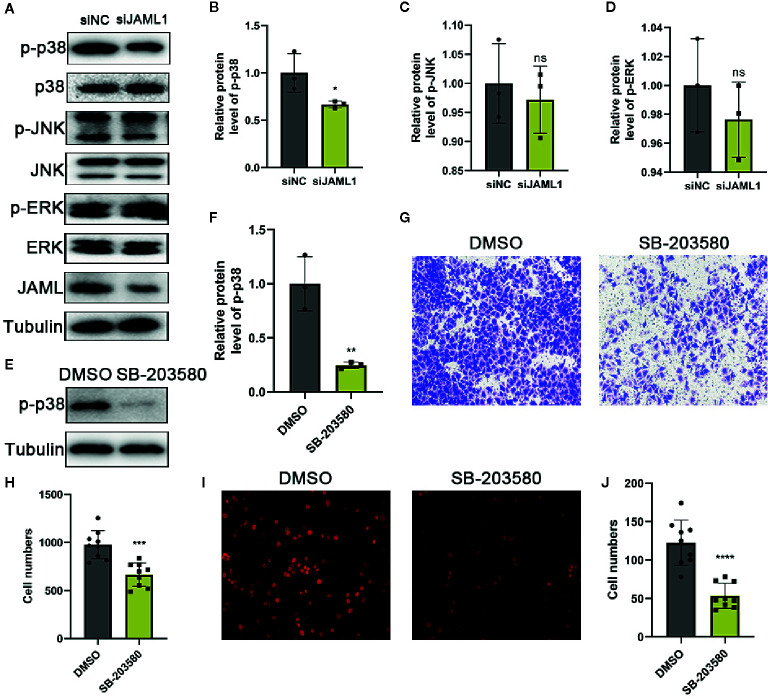
JAML promoted GC cell migration and proliferation by activating p38 signaling pathway. **(A)** The effect of JAML silencing on the phosphorylation of p38, ERK and JNK. **(B–D)** Quantitative analysis of **(A)** n=3, unpaired t test, *P < 0.05, compared with siNC group; ns, *P* > 0.05, compared with siNC group. **(E)** The effect of SB-203580 on p38 phosphorylation. **(F)** Quantitative analysis of **(E)** n=3, unpaired t test, **P < 0.01, compared with DMSO group. **(G)** Transwell migration assay to evaluate the effect of SB-203580 on cell migration in MKN-28 cells. **(H)** Quantitative analysis of **(G)** n=9, unpaired t test, ***P < 0.001, compared with DMSO group. **(I)** EdU incorporation assay to assess the effect of SB-203580 on cell proliferation in MKN-28 cells. **(J)** Quantitative analysis of **(I)** n=9, unpaired t test, ****P < 0.0001, compared with DMSO group.

In order to explore the underlying mechanism of JAML-mediated GC cells migration and proliferation, the activities of mitogen-activated protein kinases (MAPKs), including p38, JNK and ERK, were measured in GC cells by western blot. We found that JAML silencing significantly inhibited p38 phosphorylation, while did not affect the activities of ERK or JNK (**Figures 4A–D**). After that, we used SB-203580, a p38 inhibitor, to treat MKN-28 cells, which endogenously expresses high level of JAML. The results showed that the phosphorylation of p38 was effectively inhibited in MKN-28 cells treated with SB-203580 (**Figures 4E, F**). Then, the transwell migration and the EdU incorporation assays showed that SB-203580 significantly suppressed migration and proliferation in MKN-28 cells (**Figures 4G–J**). These results implied that the ability of JAML to promote GC cell migration and proliferation might be mediated by p38 signaling pathway.

## Discussion

Recently, the role of JAML in immune cell activation and inflammatory response has attracted researchers’ attention. JAML, a newly discovered adhesion molecule, is a secretory type I transmembrane glycoprotein. It can both mediate intercellular interactions and bind to intracellular proteins to mediate downstream signaling pathways (16, 17). In recent years, the expression and role of JAML on other cell types have also been gradually explored. It has been found that JAML can promote the adhesion of leukocytes to endothelial cells in myeloid leukemia (10). Our recent study found that JAML silencing delayed the formation of atherosclerosis in mice (14). Although studies on JAML under various pathological conditions are becoming more common, the relationship between JAML and tumor development has never been reported. In our current study, we found that JAML was upregulated in GC tissues and JAML promoted the proliferation and migration of GC cells, partially by regulating p38 activation.

To investigate the relationship between JAML and tumor development, we selected gastric tumors as the research object. First, we found JAML was significantly upregulated in GC tissues by IHC and was associated with higher tumor malignancy. This study demonstrates for the first time that JAML is highly expressed in GC tissues and might be a diagnostic biomarker in GC. Then, we performed experiments *in vitro*. By regulating the expression of JAML, we found that upregulation of JAML promoted, while JAML deficiency attenuated GC cells proliferation and migration. The bidirectional regulation of JAML in different types of GC cells confirmed this conclusion.

In addition, we also discussed the primary mechanism by which JAML promotes GC progression. We found that JAML may play a tumor-promoting role by activating the p38 signaling pathway. The p38 signaling pathway is a key signal transduction pathway by which tumor cells to sense and adapt to a variety of environmental stimuli, and it plays an important role in the occurrence and maintenance of tumors (18–21). We found that the phosphorylation level of p38 decreased significantly after JAML expression was downregulated. After treatment with p38 classic inhibitors, the proliferation and migration of MKN-28 cells decreased significantly, suggesting that JAML promoted the growth and movement of GC cells by activating p38.

## Conclusion

In summary, the present study revealed the high expression of JAML in GC, and results showed that JAML promoted GC proliferation and migration by regulating p38 pathway. Overall, the present data bring novel insights into the mechanisms by which JAML regulates GC and highlights the potential clinical significance of JAML in the pathogenesis of GC.

## Data Availability Statement

The raw data supporting the conclusions of this article will be made available by the authors, without undue reservation.

## Ethics Statement

The studies involving human participants were reviewed and approved by Jinan Central Hospital. The patients/participants provided their written informed consent to participate in this study.

## Author Contributions

MS, JY, and YS designed the study. YF, JY, MS, and YS prepared the first draft of the paper. YF, MS, and YS performed the statistical analysis of the data. YF, CC, SL, FX, SM, JL, and GZ performed the data collection. All authors contributed to the article and approved the submitted version. All authors agreed to be responsible for this work and ensure that any issues related to the accuracy and completeness of the paper are investigated and resolved appropriately.

## Conflict of Interest

The authors declare that the research was conducted in the absence of any commercial or financial relationships that could be construed as a potential conflict of interest.

## Abbreviations

CAR, coxsackie and virus receptor; GC, gastric cancer; IHC, immunohistochemical; JAML, junctional adhesion molecule-like protein; JAMs, junctional adhesion molecules; JAM-A, junctional adhesion molecule A; JAM-C, junctional adhesion molecule C; MAPK, mitogen-activated protein kinase.
